# Bacterial Cellulose/Graphene Oxide/Hydroxyapatite Biocomposite: A Scaffold from Sustainable Sources for Bone Tissue Engineering

**DOI:** 10.1021/acsami.4c17306

**Published:** 2024-12-19

**Authors:** Adam Aberra Challa, Nabanita Saha, Tanya Zhivkova, Radostina Alexandrova, Petr Saha

**Affiliations:** † Centre of Polymer Systems, University Institute, Tomas Bata University in Zlin, třída Tomáše Bati 5678, 76001 Zlín, Czech Republic; ‡ Institute of Experimental Morphology, Pathology and Anthropology with Museum, Bulgarian Academy of Sciences, Acad. G. Bonchev Street, Block 25, 1113 Sofia, Bulgaria

**Keywords:** bacterial cellulose, nanofibrous network, biocomposite, scaffold, sustainable biomaterial, bone tissue engineering

## Abstract

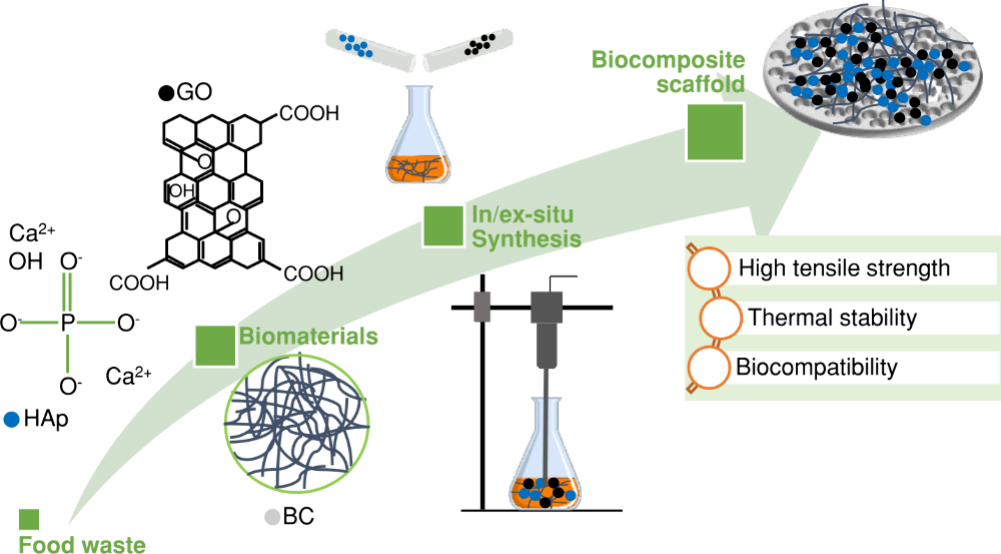

Bone tissue engineering demands advanced biomaterials with tailored properties. In this regard, composite scaffolds offer a strategy to integrate the desired functionalities. These scaffolds are expected to provide sufficient cellular activities while maintaining the required strength necessary for the bone repair for which they are intended. Hence, attempts to obtain efficient composites are growing. However, in most cases, the conventional production methods of scaffolds are energy-intensive and leave an impact on the environment. This work aims to develop a biocomposite scaffold integrating bacterial cellulose (BC), hydroxyapatite (HAp), and graphene oxide (GO), designated as “BC/HAp/GO”. All components are sourced primarily from agricultural and food waste as alternative means. BC, known for its biocompatibility, fine fiber network, and high porosity, serves as an ideal scaffold material. HAp, a naturally occurring bone component, contributes osteoconductive properties, while GO provides mechanical strength and biofunctionalization capabilities. The biomaterials were analyzed and characterized using a scanning electron microscope, a X-ray diffractometer, and a Fourier transform infrared spectrometer. The produced biocomposite scaffolds were tested for thermal stability, mechanical strength, and biocompatibility. The results showed a nanofibrous, porous network of BC, highly crystalline HAp particles, and well-oxygenated GO flakes with slight structural deformities. The synthesized biocomposite demonstrated promising characteristics, such as increased tensile strength due to added GO particles and higher bioactivity through the introduction of HAp. These inexpensively synthesized materials, marked by suitable surface morphology and cell adhesion properties, open potential applications in bone repair and regeneration.

## Introduction

Biocomposites for bone tissue engineering are currently highly researched as the need for advanced materials rises. A scaffold is required to support various cellular activities such as cell migration, proliferation, and differentiation by mimicking an extracellular matrix.^1^ Mechanically, such a material is expected to possess desirable stiffness as well as tensile and compressive strength. Moreover, the porous architecture of bone tissue repair scaffolds is characterized by four major factors: porosity, pore size, surface area, and interconnectivity.^2^ Several types of biomaterials are used as building materials to fulfill these requirements. Mainly classified as ceramics, metals, polymers, and composites, these materials are required to possess the properties mentioned above. Composite scaffolds are preferred when it comes to integrating various properties that a biomaterial needs in this sector. The desired surface properties, mechanical stability, chemical structure, and biological cues can be tailored in the making of composite biomaterials.^2^ For instance, polymers are highly regarded as viable materials for tissue engineering because of their biocompatibility and close structure to the extracellular matrix of cells. Although polymeric biomaterials typically exhibit favorable ductility, if unreinforced, their inherent mechanical properties often fall short of the stiffness required for load-bearing bone substitute applications. Polymer-based composites address this limitation by offering a wider range of tailorable properties, enabling the design of implants with enhanced mechanical performance for bone repair.^3^

One such biopolymer is bacterial cellulose (BC), known for its biocompatibility. Due to this and other physicochemical properties, it is widely used for wound healing, tissue engineering, and drug delivery.^4^ It can also be functionalized to have antibacterial properties.^5^ Furthermore, due to its fine fiber network, good biocompatibility, and high water-holding capacity, BC is one of the most widely used biomaterials for making scaffolds. The intricate detail of the nanofibril network of BC gives rise to its high surface area and porosity, which makes it ideal for cell growth and propagation, a necessary attribute for a good tissue scaffold.^6^ In addition, the chemical network of BC proves its ability to form biocomposites. Given the abundant hydroxyl groups on its surface, strong hydrogen bonds create the necessary attachment of BC to other biomaterials.^7^

Hydroxyapatite (HAp), naturally found as part of the bone in vertebrates, is another crucial biomaterial in extended use. It is a calcium phosphate compound [Ca_10_(PO_4_)_6_(OH)_2_)] known for its biocompatibility, osteoconductive properties, and proliferation of osteogenic cells.^8^ Due to these attributes, HAp is considered a suitable biomineral for bone replacements, grafts, and tissue engineering. However, its application is limited by its lack of mechanical properties, including low fracture toughness, reduced tensile and compressive strength, and inherent brittleness.^9^ This is mitigated by the synthesis of composites of HAp through substitution of its ions.^10^ This ability to bind with other polymers introduces further advantages such as interfacial adhesion between reinforcement and polymers, enhancing thermal stability and creating pores in a polymer matrix for the growth of tissues in regenerative applications.^11,12^ Various biomaterials have been used to form a HAp biocomposite based on the desired synthesized material and intended application.

Graphene oxide (GO) is a form of graphene with excellent properties such as electrical conductivity, mechanical strength, and optical properties.^13^ Because of its oxygen-containing functional groups, GO is easily biofunctionalized, which prompts its use for biomedical fields.^14^ However, the production of GO poses a concern. The conventional method of synthesizing GO requires graphite powder/flakes. This is a material either sourced naturally, which involves large industrial procedures, or produced synthetically, both of which require very high energy.^15^ To solve this problem and ensure its use as a biomaterial, several alternative sources are being utilized.

To combine the issues of finding alternative sources for the synthesis of the above-mentioned components and to benefit from their vital properties, several studies are being undertaken to find alternative sources for the above components. BC has been cultivated in a whey medium,^16^ liquid tapioca waste,^17^ or citrus peel.^18^ Substitute calcium sources such as animal bones, shells, several plant leaves, or algae have been utilized to synthesize HAp,^19^ whereas GO has been synthesized from carbon sources such as palm kernel shells and empty fruit bunch,^20^ rice husk and coconut shells,^21^ or coffee grounds.^22^

There have been some studies made to form biocomposites that involve the three materials mentioned above in combination or with other polymers. For instance, a GO/HAp/cellulose nanocomposite was prepared for antimicrobial properties by Yahia et al.^23^ Ag nanoparticles were used to immobilize the composite. In turn, for deposition of these particles, GO played a role through electrostatic forces. As a result, the antimicrobial property of the composite was highly effective on both Gram-positive and Gram-negative bacteria. In another research, scaffolds of HAp nanoparticles with reduced GO were synthesized to promote the regeneration of bone tissues in a defected cranium of rats.^24^ Increased bone density and osteogenic mineralization of rat bone marrow stem cells indicated the effectiveness of the scaffolds in bone growth and the treatment of tumors. In contrast, Umar Aslam Khan et al. produced a scaffold made of BC and β-glucan for bone tissue engineering purposes.^25^ They incorporated HAp and GO to reinforce the scaffolds. The result was a mechanically strong biomaterial with suitable surface morphology and structure, which ensured the viability of mouse osteoblast cells. Cell adhesion to the scaffolds and antimicrobial properties against selected pathogens were enhanced with an increased GO amount.

This research focuses on synthesizing a biocomposite from BC, HAp, and GO for bone tissue engineering. To our knowledge, we have not found any research that dealt with such a composite, where all of the components were sourced mainly from agricultural and food waste alternatives. It aims to substitute the traditional sources of obtaining the materials and, hence, reduce the overall energy required to produce the composite. The biocomposite was characterized for its desirable properties and its biocompatibility.

## Experimental Section

### Materials

To produce BC, HAp, and GO, apple juice made from waste apple fruits collected in Zlin, Czech Republic, egg shells taken from a personal kitchen, and coffee waste obtained from spent Arabica coffee grounds were used, respectively. Laboratory-grade chemicals iron(III) chloride hexahydrate (FeCl_3_·6H_2_O), nitric acid (HNO_3_), 98% pure sulfuric acid (H_2_SO_4_), sodium nitrate (NaNO_3_), potassium permanganate (KMnO_4_), sodium hydroxide (NaOH), 30% pure hydrogen peroxide (H_2_O_2_), hydrochloric acid (HCl), and phosphoric acid (HPO_4_) were purchased from Sigma-Aldrich, Czech Republic.

To produce BC, a *Komagataeibacter xylinus* bacterial strain was obtained from the Czech Collection of Microorganisms, Brno, Czech Republic. Agar, citric acid, sodium phosphate dibasic dodecahydrate (Na_2_HPO_4_·12H_2_O), peptone, and yeast extract were purchased from Sigma-Aldrich, Czech Republic. For the biocompatibility study, the Saos-2 permanent cell line from human osteosarcoma was provided by the Institute of Experimental Morphology, Pathology and Anthropology, Bulgarian Academy of Sciences. Fetal bovine serum (FBS) and Dulbecco’s modified Eagle medium (DMEM) were obtained from Gibco-Invitrogen (U.K.). Dimethyl sulfoxide (DMSO) and trypsin were purchased from AppliChem (Darmstadt, Germany). Thiazolyl blue tetrazolium bromide was purchased from Sigma-Aldrich Chemie GmbH (Germany). The antibiotics (penicillin and streptomycin) for cell cultures were from Lonza (Belgium).

### Methods

BC was produced in growth media with a 1:1 ratio of Hestrin–Schramm (HS) and apple juice. The HS medium contained 20 g/L glucose, 5 g/L yeast extract, 5 g/L peptone, 2.7 g/L Na_2_HPO_4_·12H_2_O, and 1.15 g/L citric acid. To activate the bacteria, 5 loops of the bacterial strain were inoculated into 5 mL of HS media. After 3 days of incubation, this was added to 100 mL of prepared HS/apple juice growth media. This was, in turn, incubated at 30 °C for 15 days. Purification of the produced BC mats was performed by washing them with distilled water first and boiling them with a 0.5 N NaOH solution at 80 °C for 1 h. Then a neutral pH was achieved by repeated washing with deionized (DI) water.

HAp was synthesized from egg shells as the calcium source and phosphoric acid as the phosphorus source. Egg shells were washed thoroughly and dried in an oven overnight. Afterward, they were ground in a mortar and pestle, followed by combustion in a tube furnace at 1000 °C for 2 h. The obtained calcium oxide sample was dissolved in DI water to form Ca(OH)_2_. Ca(OH)_2_ (0.5 M) was then mixed with H_3_PO_4_ (0.3 M), where the latter was added in a dropwise manner at 2 mL/min to achieve the required stoichiometric ratio. This mixture was allowed to age for 48 h and filtered afterward. The filtrate was then washed with DI water to remove any residuals. After this sample was dried in an oven overnight, it was calcined in a tube furnace at 700 °C for 2 h to finalize the crystallization.

GO was prepared from spent coffee grounds. The raw sample was thoroughly washed and soaked in FeCl_3_·6H_2_O for 24 h for graphitization purposes. This was dried overnight until the moisture was completely gone. The sample was then carbonized in a tube furnace at 1000 °C for 2 h. Afterward, concentrated HNO_3_ was used to remove the iron salt from the carbonized material. This mixture was then washed with DI water. The graphitized carbon ash was then oxygenized using the modified Hummer’s method, as mentioned in our previous paper.^22^ Briefly, 2 g of graphitized carbon was added to 50 mL of H_2_SO_4_. The mixture was put in an ice bath and stirred, keeping the temperature below 10 °C. A total of 1 g of NaNO_3_ was added and stirred for 2 h. Then, 6 g of KMNO_4_ was added, and the temperature was raised to 35 °C and stirred for 2 h. DI water was then added, simultaneously increasing the temperature to 90 °C. After 1 h, 200 mL of DI water and 10 mL of H_2_O_2_ were added to kill the reaction and remove the residual products of KMNO_4_. After filtration, it was then mixed with concentrated HCl and centrifuged. The supernatant was decanted, and the rest was washed several times with DI water. Finally, ultrasonication took place to obtain the final GO particles.

Three methods were used to produce the BC/HAp/GO biocomposites.

#### Method I (In Situ)

A GO solution (1 mg/mL) was added to the growth media after some BC mat was formed (after the seventh day of incubation), and BC was left to grow in the modified media for 7 more days. The final mat was named BC/GO. Similarly, BC/HAp mats were produced in a similar procedure by adding a HAp solution (2 mg/mL) instead of GO.

#### Method II (Ex Situ)

After the BC pellicles were formed and purified, they were immersed in a HAp/GO solution (with each having a concentration of 2 mg/mL) overnight. This mixture was then ultrasonicated in a probe sonicator for 30 min.

#### Method III (Ex Situ)

The purified BC pellicles were ground and homogenized. A total of 25 mL of a HAp/GO suspension was added, and the mixture was ultrasonicated. This was left to mineralize for 24 h, after which it was cast onto plates.

All BC mats and composites were freeze-dried for further processes.

### Characterization and Testing

The morphology of the surface of the samples was checked using scanning electron microscopy (SEM; FEI, Brno, Czech Republic) with energy-dispersive X-ray (EDX) spectroscopy, which was used to identify the elements present. The samples were attached to double-sided carbon tape and sputter-coated with Au–Pd particles prior to the microscopy. The chemical groups present in the particles and scaffolds were identified using Fourier transform infrared (FTIR) spectroscopy in attenuated-total-reflectance mode (Nicolet iS5, Thermo Scientific, USA). The scanning frequency range was 4000–400 cm^–1^. To identify the crystallinity and diffraction patterns, an X-ray diffractometer (MiniFlex 600, Rigaku, USA) was utilized. The data were obtained at 40 kV and 15 mA using Co Kα radiation (wavelength of 1.79 Å). 2θ values were read in the range between 4° and 80°. The data were then converted to Cu Kα radiation using a PowDLL converter, version 2.911.0.0. Brunauer–Emmett–Teller (BET) analysis was used to analyze the surface area and porosity of the samples. This was performed using a surface area analyzer (BELSORP-mini II, BEL Japan, Inc., Japan). The samples were analyzed under a nitrogen atmosphere (adsorption–desorption isotherms were read at 77 K). To determine the thermal stability of the samples, thermogravimetric analysis (TGA) was performed using a TGA Q500 device (TA Instruments, USA). The tests took place under a nitrogen atmosphere in a temperature range of 25–800 °C and at a heating rate of 10 °C/min. Tensile tests of prepared scaffolds were conducted by using a M350-5CT machine (Testometric, U.K.). Strips were prepared in rectangular dimensions of 10 × 40 mm with a clear spacing between the attachment clamps of 2 cm. The thickness of the strip was measured by using a digital thickness gauge (Mitutoyo, Germany). A cross-head speed of 10 mm/min was maintained using a 1 kg load cell. The tensile stress and strain at the breaking point of the strip were recorded. The apparent Young’s modulus was defined by the slope of the linear region of the strain–stress curve during the stretching stage. Five replicates were conducted for each sample.

### Biocompatibility Study

The prepared biomaterial scaffolds were tested indirectly for biocompatibility. The materials were cut and placed in 6-well culture plates and subjected to UV sterilization. Drops of 10 μL of FBS were added at 30–32 °C to adhere the sample to each well surface. Subsequently, 2.5 mL of DMEM [enriched with 10% FBS and antibiotics (100 U/mL penicillin and 100 g/mL streptomycin)] was added to the wells. The same was done to wells not containing the scaffold materials, which served as controls. The setup was then incubated in a humidified atmosphere at 37 °C with 5% CO_2_ in air for 1, 3, and 5 days. The cell culture medium (including sample extracts and a control medium) was harvested and used in subsequent indirect experiments.

For the cell viability/proliferation study, human osteosarcoma Saos-2 cells were plated in 96-well flat-bottomed microplates at a concentration of 1.0 × 10^5^ cells/well in fresh DMEM supplemented with 10% FBS and antibiotics (as was described). After 24 h, the culture medium was removed from each well and replaced with 100 μL of DMEM containing sample extracts obtained after 1-, 3-, and 5-day incubation periods or the control medium.

The effect of the compounds on the cell viability/proliferation was evaluated after 72 h using a 3-(4,5-dimethylthiazol-2-yl)-2,5-diphenyltetrazolium bromide (MTT) colorimetric test. The method consisted of 3 h of incubation with a MTT solution (5 mg of MTT in 10 mL of DMEM) at 37 °C in a CO_2_ incubator (5% carbon dioxide and 95% air), followed by extraction with a mixture of absolute ethanol and DMSO (1:1, v/v) to dissolve the blue formazan. Extinction measurement was preceded by a 10 min shaking (Titertek shake machine, Flow Laboratories) of the microplates and conducted by an ELISA automatic microplate reader (TECAN, Sunrise, Austria) at a wavelength of 540/620 nm optical density. The relative cell viability, expressed as a percentage of the control (100% viability), was calculated for each period in which the materials were incubated in the medium. The results obtained were graphically represented.

## Results and Discussion

The first part of this study dealt with synthesizing BC, HAp, and GO from their respective alternative sources. BC was produced in a medium containing apple juice, aimed at reducing the sole use of the conventional HS medium, which can be expensive, especially for industrial and large-scale production of BC.^5^ However, the sugar content in apple juice (mainly fructose and sucrose) is not sufficient to provide all of the nutrients needed for the formation of cellulose fibers.^26^ Thus, additional glucose and nitrogen sources in the form of peptone and yeast extract were provided by modification with a HS medium. Adding HAp and GO solutions in the growth media during the in situ synthesis was undertaken under static conditions. Even though agitated conditions would result in a better suspension of the particles, the static method was chosen to avoid the disintegration of the BC pellicles. As a result, the biocomposites obtained were full-bodied BC mats. They had average thicknesses of 2.5 mm just after synthesis.

### Morphological, Physical, and Chemical Characteristics

**Figure 1:**
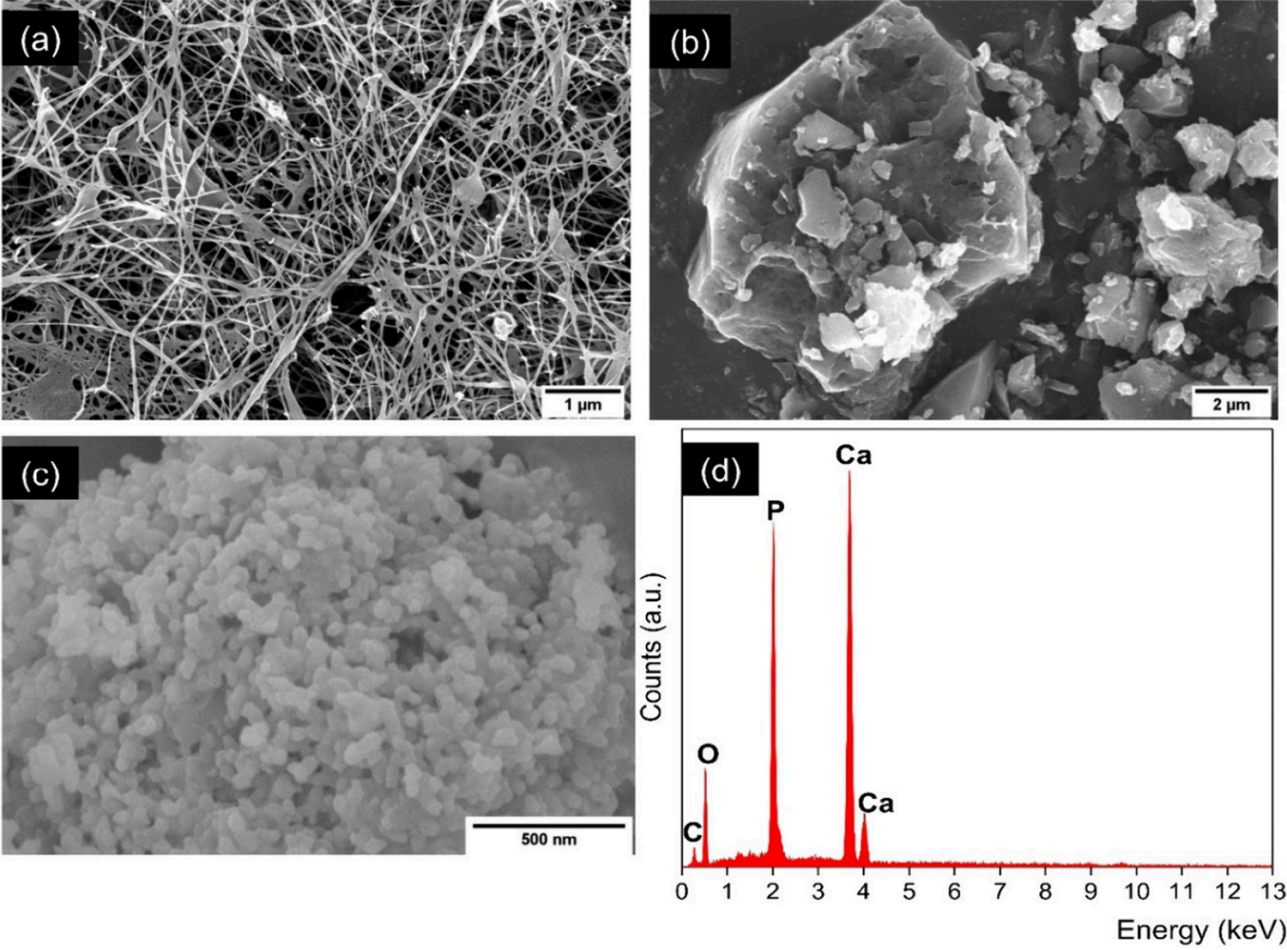
SEM micrographs of (a) a dried BC mat, (b) GO flakes, and (c) HAp powder and (d) EDX spectrum of HAp nanoparticles.

Prior to the morphological analysis of the BC mats, they were subjected to freeze-drying. The surface characteristics were then checked using SEM. Upon the investigation of pure BC, a well-networked system of nanofibers with ample interconnection can be seen in Figure 1a. The fibril network is randomly arranged and is a result of inter- and intramolecular interactions between β-(1,4)-glucan chains through hydrogen bonding.^27^ Sufficient porosity, which is a highly sought-after property as a biomaterial, can also be observed. GO, after the processes of intercalation and oxygenation, resulted in flat surfaces showing the exfoliated layers of graphitized carbon, as can be seen in Figure 1b. The irregularity in the flakes is a result of being sourced from a biomass precursor.

Upon investigation of the morphology of the synthesized HAp, rod-shaped nanoparticles were observed, as shown on the SEM micrographs in Figure 1c. After the second heat treatment, the smaller nanoparticles merged to form macroparticles. It also resulted in a more regular shape. This phenomenon can be associated with a recrystallization phase at high temperatures.^28^ The Ca/P ratio was calculated by observing three EDX spectra of the HAp particles (a representative spectrum is shown in Figure 1d). The average Ca/P ratio was calculated to be 1.72. This is very close to 1.67, which is the stochiometric Ca/P ratio of pure HAp.^28,29^ The increase in the Ca/P ratio through the combined calcination and precipitation synthesis of HAp can be attributed to the probable presence of CaO in the final product.^19^

**Figure 2:**
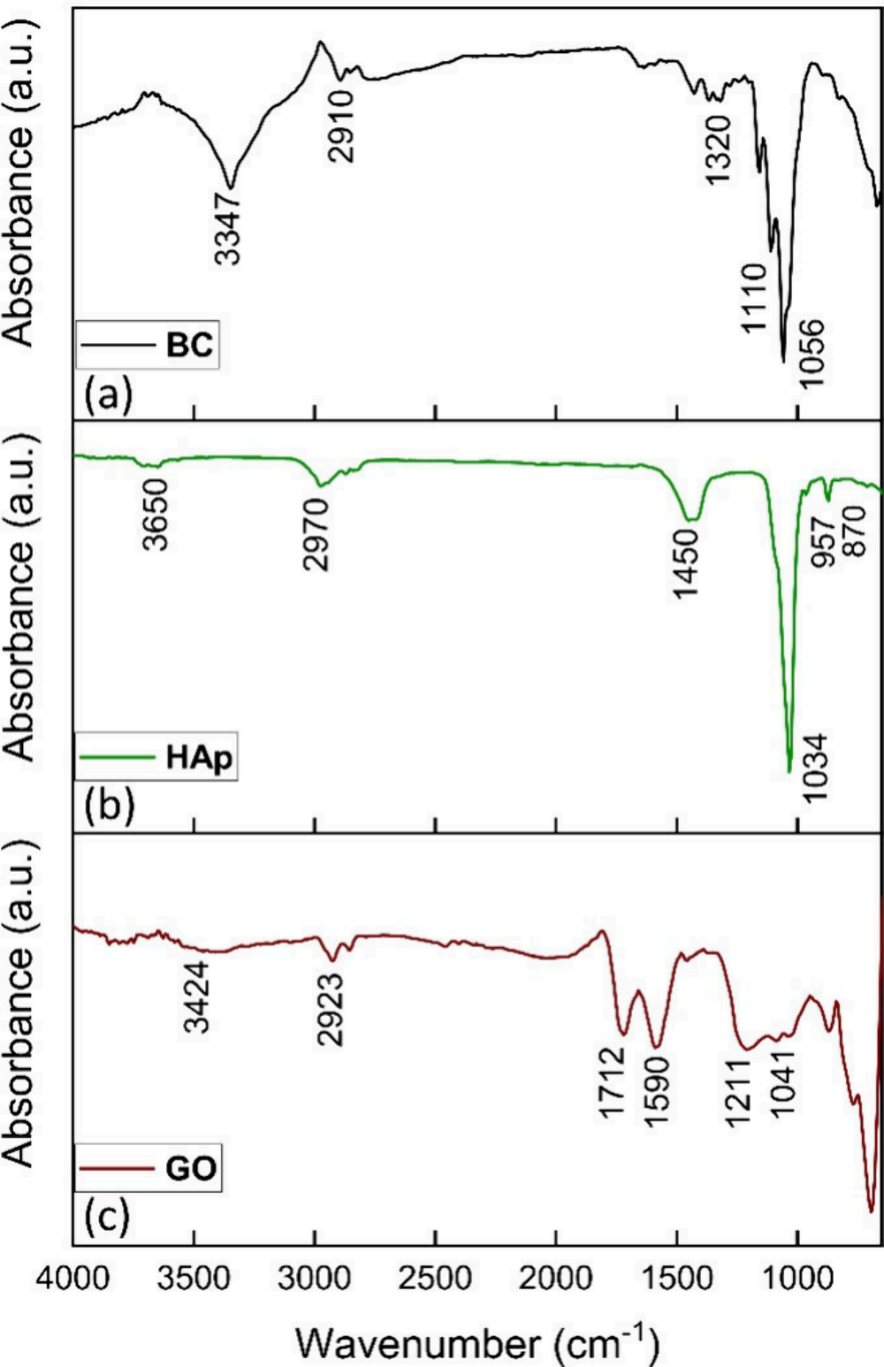
FTIR spectra of (a) a BC mat, (b) HAp powder, and (c) GO particles.

The different chemical groups found in each of the biomaterials were identified by using a FTIR spectrometer. BC exhibits its expected functional groups in its FTIR spectra. Figure 2a shows stretching vibrations of hydroxide (OH) and C–H at 3347 and 2910 cm^–1^, respectively, and a bending vibration of C–H at 1320 cm^–1^.^30^ In addition, the spectra identified an asymmetric C–O–C stretching vibration at 1110 cm^–1^ and a C–O stretching frequency of the β-(1–4)-glycosidic links at 1056 cm^–1^.^31^ In HAp, the main chemical groups are the phosphate and carbonate groups. In Figure 2b, the spectra show the expected carbonate (1450 and 870 cm^–1^, indicating the stretching and bending vibrations of CO_3_, respectively) and phosphate (1034 and 962 cm^–1^, showing the bending and stretching vibrations of PO_4_, respectively) groups.^32^ A stretching mode of the hydroxide (OH) groups was also observed at 3650 cm^–1^. In contrast, GO is a compound of carbon. Hence, Figure 2c shows the different carbon and oxygen functional groups that it entails. The peak at 3424 cm^–1^ can be attributed to the stretching frequency of hydroxyl group (OH).^33^ The expected stretching vibrations of the carbonyl group (C=O) at 1712 cm^–1^, C=C at 1590 cm^–1^, and epoxy group (C–O–C) at 1041 cm^–1^ were also observed. In addition, the C–O stretching from carboxylic groups appeared at 1211 cm^–1^, whereas the asymmetric stretching vibration of C–H was seen at 2923 cm^–1^.^34^

**Figure 3:**
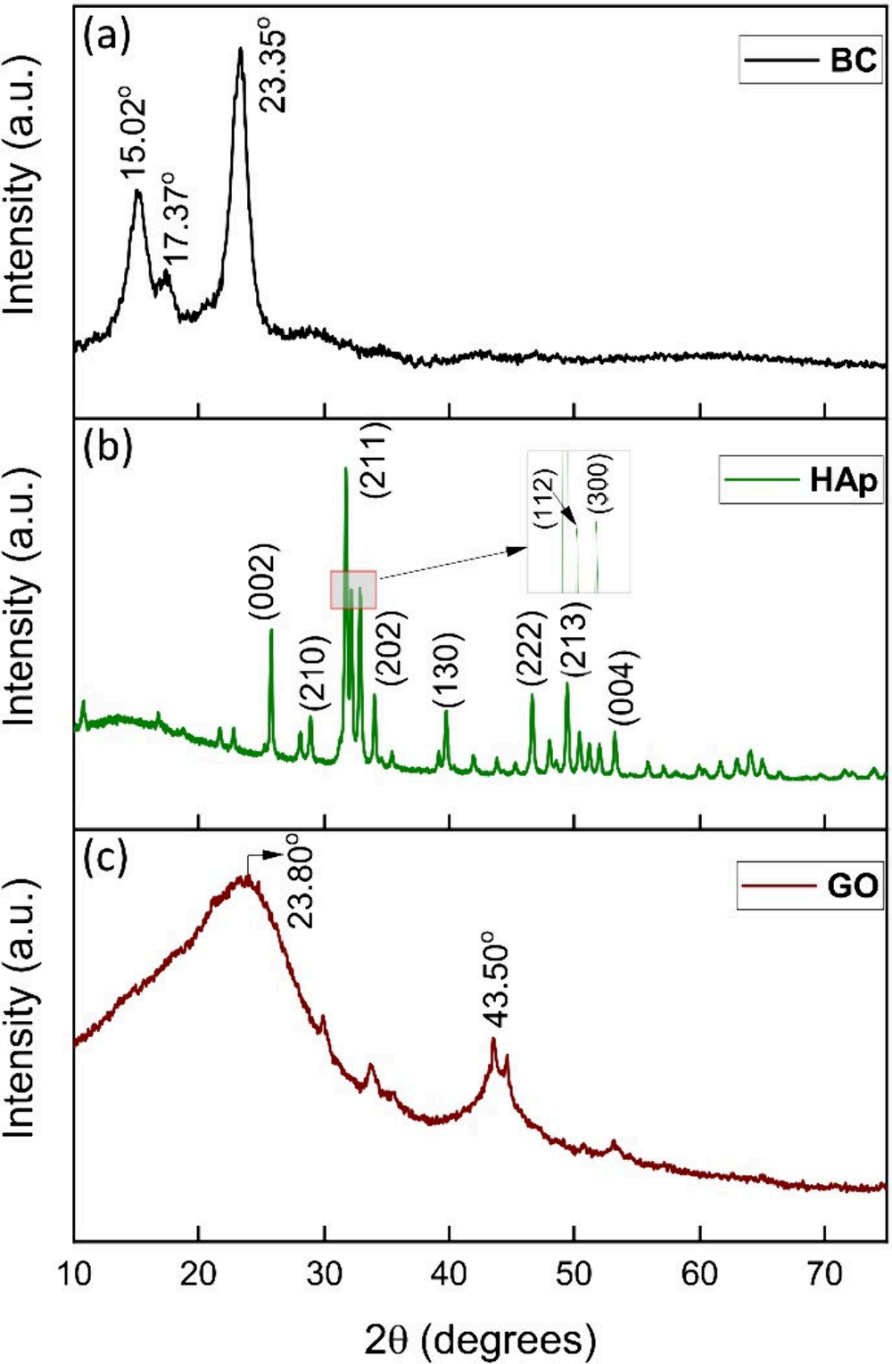
XRD spectra of (a) a BC mat, (b) HAp powder, and (c) GO flakes.

The crystallinity of the biomaterial components was studied by using X-ray diffraction (XRD). The typical diffraction pattern of BC can be seen in Figure 3a. The three main diffraction peaks at 2θ values of 15.02°, 17.37°, and 23.35° correspond to the (110), (110), and (200) planes of cellulose I_β_, respectively.^35^ These sharp peaks indicate completion of the final crystallization of cellulose fibrils after the formation of β-(1,4)-glucan chains.^36^ Similarly, the crystalline nature of the synthesized HAp was expressed in the sharp peaks (Figure 3b), mainly those that correspond to the (211), (112), and (300) planes, identified using ICSD card 01-080-6199. These Miller indices represent a hexagonal crystal system with cell parameters *a* (9.421), *b* (9.421), and *c* (6.882). Using Scherrer’s equation, the crystalline size was calculated as 36 nm, matching the expected figures. GO showed a broader diffraction peak at 23.8° (Figure 3c), indicating the existence of amorphous carbon particles. Because the ordered graphite structure of natural graphite is lacking in the case of a biomass carbon precursor, low crystallinity is evident. This structural property is evident in the reports of studies made by other researchers who synthesized GO from biomass such as coconut shells, rice husk, and bagasse.^37^

**Figure 4:**
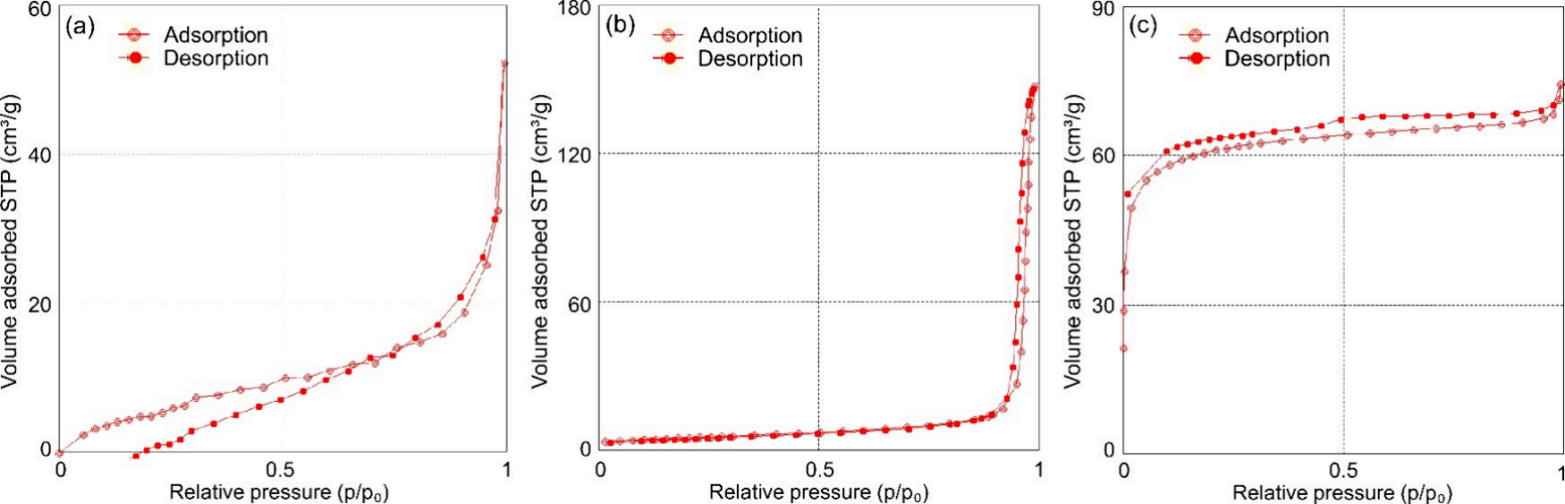
Nitrogen adsorption–desorption isotherms for (a) BC nanofibrils, (b) HAp powder, and (c) GO particles.

The surface area of the individual components was investigated by using nitrogen adsorption–desorption measurements (Figure 4). The results of the surface area, pore diameter, and pore volume measurements are summarized in Table 1. The adsorption–desorption curve for BC in Figure 4a shows a type IV-A isotherm in the presence of a hysteresis loop. This, coupled with the measured surface area of 22 m^2^/g, reflects the typical characteristics of pristine BC pellicles.^38^ The curve for HAp particles shown in Figure 4b is of a type IV isotherm with an H3 hysteresis loop, which matches the results for eggshell-derived HAp.^39^ The pore diameter of 51.3 nm can be classified as macropores according to IUPAC classifications. Similarly, the adsorption/desorption isotherm of GO shown in Figure 4c can be assigned as a combination of type I and IV curves with a hysteresis loop measured between the relative pressure range values of 0 and 1.^40^ This represents the presence of interconnected micro- and mesopores. The calculated pore diameter of 1.98 nm proves such a presence. Furthermore, this phenomenon is associated with the high specific surface area of GO registered as 226 m^2^/g. This value is lower than reported values for conventional GO; however, the surface area and pore diameter are comparable to or higher than the respective values reported by researchers who used alternative sources to synthesize GO.^41,42^ From the values discussed above, it can be inferred that the combined effect of the high surface area of GO and the highly porous structure of HAp will be a desired structural input during formation of the BC composite.

**Table 1:** Values of the BET Measurement Results for BC, HAp, and GO Particles

sample	surface area (m^2^/g)	pore diameter (nm)	pore volume [cm^3^(STP)/g]
BC	22.05	13.57	5.0664
HAp	17.61	51.3	4.05
GO	226	1.98	51.942

### Biocomposite Fabrication

**Figure 5:**
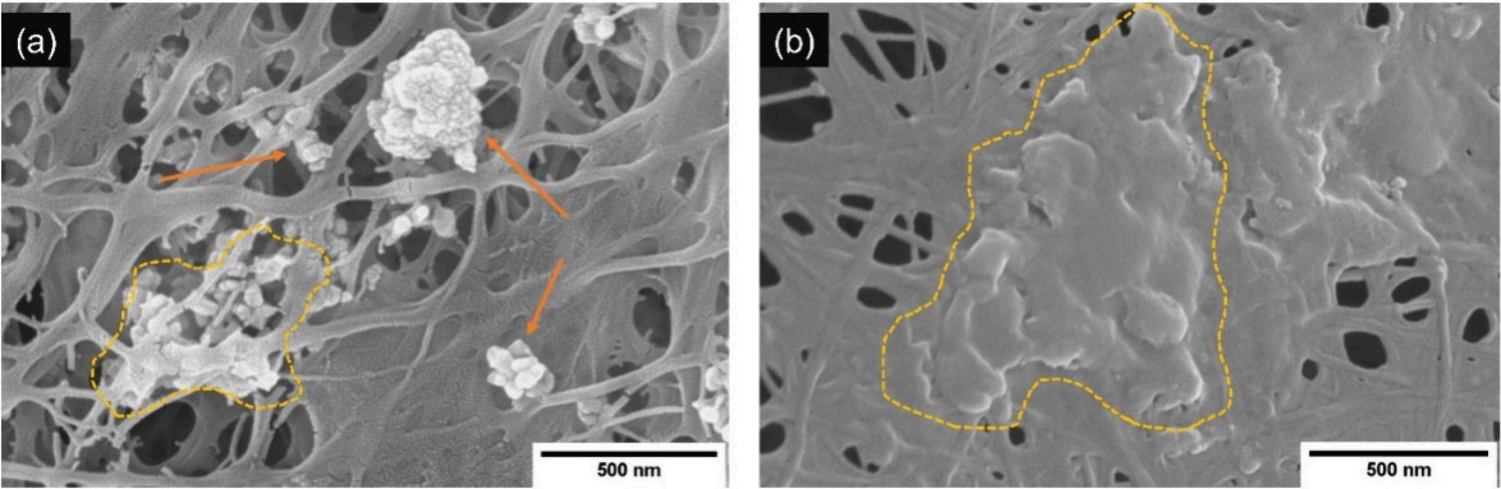
SEM micrograph images showing the incorporation of fillers in the BC matrix: (a) HAp particles indicated by orange arrows and agglomerated particles highlighted by a yellow line; (b) embedded GO particles encircled by a yellow line.

As described in the Methods section, three methods were adopted for the fabrication of the BC/HAp/GO biocomposite. The first method (Method I) was in situ synthesis. This approach entailed adding a filler-containing suspension to the growth medium of BC once a BC surface was formed. The rest of the BC membrane was allowed to grow with the incorporated particles. This method yielded a layer of BC with minimal attachment of the added nanoparticles (suspensions of HAp and GO). This was due to settlement of the particles in the static culture. It was also observed that, after the addition of the suspensions, a new layer of BC formed above with minimum attachment or no attachment to the previous layer, because the latter was pushed to the subsurface. Moreover, after purification of the pellicles, the nanoparticles were removed and remained only on the outermost edges of the pellicles. However, in the areas where the particles were incorporated into the BC matrix, there was a good blend between the nanofibrils and particles due to the porous nature of BC. In Figure 5a, HAp particles are seen embedded in the nano- and micropores of the BC films, whereas in Figure 5b, the GO layers appear to seamlessly integrate with the BC surface.

**Figure 6:**
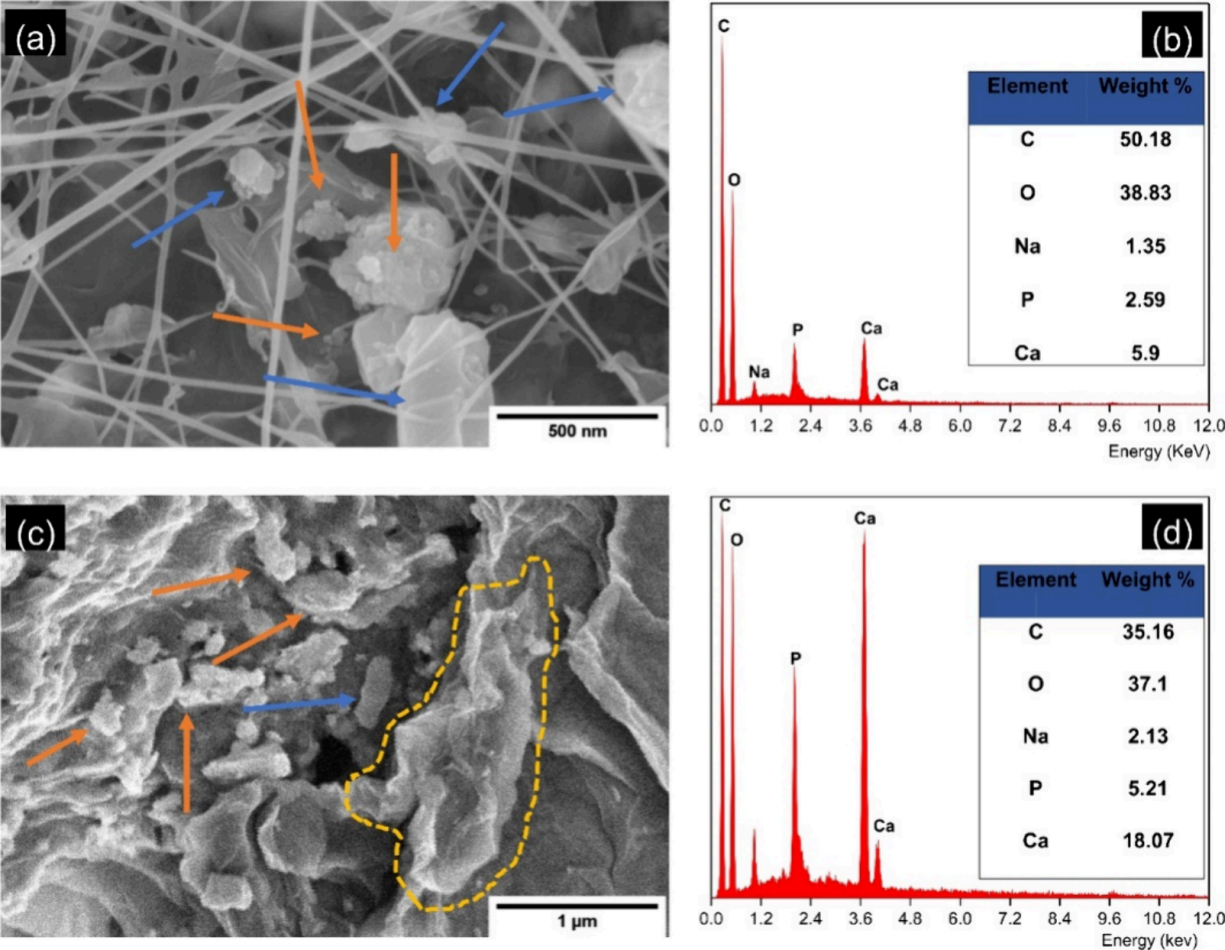
SEM micrograph images showing the assembly of a BC/HAp/GO composite through ex situ synthesis following Methods II (a) and III (c) (HAp particles are indicated by orange arrows, whereas GO particles are shown by blue arrows and yellow lines). Corresponding EDX spectra of Methods II (b) and III (d).

On the other hand, two methods were used to synthesize BC/HAp/GO composite ex situ. The first involved immersing the purified BC mat in a HAp/GO suspension, followed by ultrasonication (Method II). The second method (Method III) consisted of blending and homogenizing the BC pellicles and mixing the output with a HAp/GO suspension by ultrasonication, followed by casting and freeze-drying (Method III). In Figure 6a, an SEM image of the former shows that the filler nanoparticles are well mixed in the cellulose fibrils. The intramolecular bonds were intact and induced an overlapping and layered structure between the cellulose fibers without breaking the structure of the network. Conversely, the surface of the latter composite, marked by the image in Figure 6c, is observed to have a completely new morphology with ample presence of HAp and GO particles. The agglomerated HAp structure is evident, as indicated by the orange arrows, whereas the layered surface of GO is seen to blend into the BC film (highlighted by the yellow enclosure). The EDX spectra, as shown in Figure 6b,d for Methods II and III, respectively, clearly highlight the presence of carbon elements from the GO and Ca/P components of the HAp particles. The homogenized BC composite is seen to have a higher presence of Ca and P ions compared to that of the former synthesis method.

The pore sizes of the scaffolds were observed to differ between the in situ and ex situ synthesis methods. In the in situ method, the pore sizes resembled the native BC network due to minimal physical intervention, which preserved the inherent porosity. In contrast, the ex situ method resulted in larger micropores, highlighting the role of ultrasonication in altering the scaffold structure. Additionally, the incorporation of HAp particles further contributed to expansion of the pores.^43^ This phenomenon, combined with the biocompatible nature of HAp, is expected to support cell attachment and proliferation, as demonstrated in a later section of this paper. Ideal pore sizes ranging from 50 to 500 μm have been reported to facilitate cell attachment, nutrient transport, and subsequent vascularization during tissue regeneration.^2,44^ Despite this, the present pore distribution contributed to improved mechanical strength and supported cell proliferation, as will be shown in subsequent sections.

**Figure 7:**
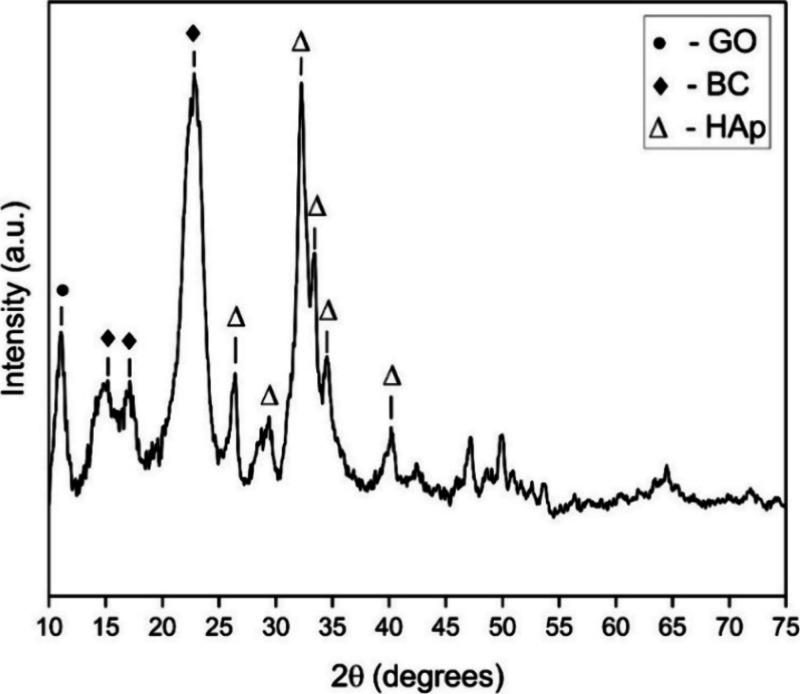
XRD spectrum of an ex situ synthesized BC/HAp/GO composite.

To further investigate the appropriate incorporation of the filler components into the biocomposite, the following XRD spectra can be seen. In Figure 7, the distinctive peaks of BC at 15.2°, 17.1°, and 22.8° are evident, which shows the undisturbed crystallinity of BC. The (211), (112), (300), and other planes of HAp particles are also represented at their respective diffraction angles. Additionally, the (002) plane of GO nanoparticles is identified by the peak at 11.05°.

This further proves that the crystallinity of the BC structure was not affected by the addition of filler particles.

### Thermal Stability

**Figure 8:**
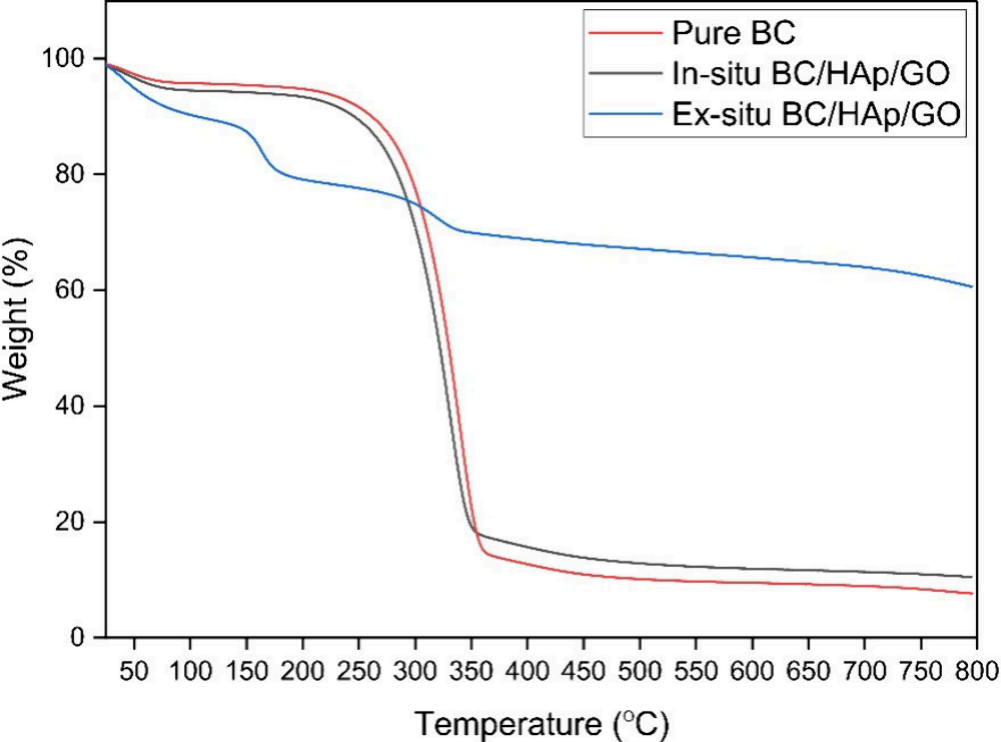
TGA results of synthesized pure BC and its composites.

TGA is commonly conducted to determine the thermal stability of materials for a given temperature range. It helps to identify the decomposition mechanism of organic matter and the presence of strong bonds within the material’s structure that degrade at higher temperatures. As is evident from Figure 8, BC exhibited a minimal weight loss (∼5%) until 200 °C, which can be interpreted as the evaporation of trapped moisture. Significant degradation (approximately 83%) occurred around 292 °C, attributed to depolymerization and decomposition of the glycosidic bonds.^38^ The final stage of degradation took place after 350 °C, which is associated with the formation of char residue. The in situ produced biocomposite showed a decomposition pattern similar to that of pure BC. This can be explained by the minimal retention of HAp/GO particles in the BC network after purification. Nevertheless, the second stage of weight loss decreased to 77.5%, indicating a slight improvement in thermal stability. In contrast, the highest thermal stability was exhibited by the ex situ prepared biocomposite (Method II). The initial weight loss up to 150 °C is associated with the loss of oxygen-containing functional groups of GO and the moisture in BC. Subsequent weight loss between 170 and 340 °C can be attributed to BC degradation, although the extent of degradation was significantly reduced. This improvement can be ascribed to the strong hydrogen-bond formation between the filler nanoparticles and BC fibrils, minimizing the exposure of the glycosidic bonds to thermal breakdown.

As a highly crystalline bioceramic, HAp significantly contributed to the improved thermal stability of the scaffold. The physical cross-linking within the composite enhanced functionalization of the BC matrix and restricted its chain mobility, further improving thermal resistance.^45^ At 800 °C, the resulting mass residue of the Method II biocomposite was 60%. In comparison, that of pure BC was 8%. This indicates that approximately 52% of the composite’s mass is attributable to the mineral components within the BC matrix.

### Mechanical Properties

**Figure 9:**
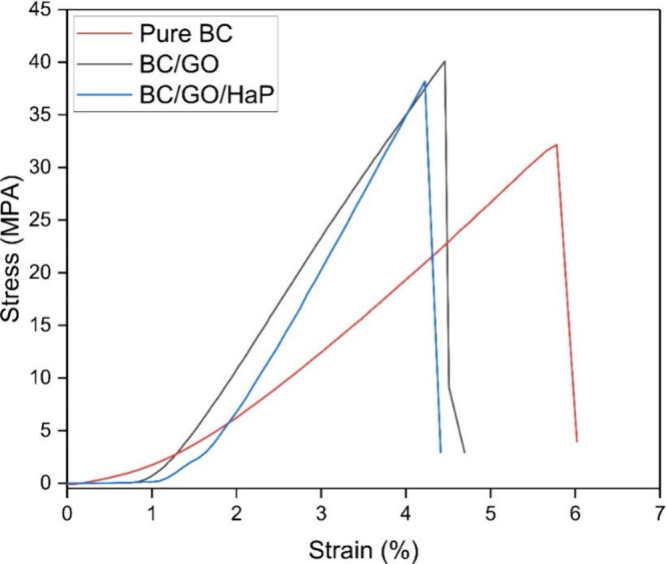
Tensile stress–strain graph of pure BC and its composites.

Tensile strength tests were conducted to investigate the value of incorporating GO particles in the biocomposite because they are known to contribute toward improved mechanical properties. Strips of pure BC, BC, and GO, and ex situ prepared BC/HAp/GO composites, were tested. With a clear distance of 20 mm between the grips, a load cell of 1 kg was used to exert the tensile force. As can be seen in Figure 9, the highest average tensile stress was obtained for scaffolds containing GO. The BC/GO sheets demonstrated a 24.73% increase in tensile strength compared to pure BC strips, while the BC/HAp/GO sheets showed an 18.74% increase. These gains, due to the addition of GO particles, match findings from other studies.^46,47^ This phenomenon could be associated with the interaction between GO and the cellulose matrix through hydrogen-bond formation.^48^ However, the incorporation of HAp particles into the BC/HAp/GO composites did not result in a further increase in mechanical strength, despite the known capacity of GO and HAp molecules to form a strong matrix. This limitation may be attributed to the physical cross-linking strategy employed for HAp and GO after their separate synthesis. HAp was synthesized from calcium and phosphate precursors and thermally treated to achieve its crystalline form—a process that could not be achieved through in situ synthesis (i.e., directly adding the precursors to a GO-containing solution). This approach may have resulted in a lower-than-expected mechanical strength, possibly due to the incomplete nucleation of calcium ions. An alternative strategy could involve the in situ synthesis of HAp particles using chemical cross-linking. However, this method was deliberately avoided to minimize the potential toxicity of the scaffolds.

Table 2 summarizes the Young’s modulus, strain, and elongation at break for the specimens. BC/GO has the highest Young’s modulus, indicating the strong bond between GO and BC networks. However, the strain at break decreased by the addition of GO particles. This shows the slightly ductile characteristics of BC membranes and the brittle nature of GO nanosheets.^49^

**Table 2:** Values for the Mechanical Properties of Pure BC and Its Composites

sample	Young’s modulus (MPa)	strain at break (%)	elongation at break (mm)
BC	87.46	5.78	1.156
BC/GO	118.46	4.46	1.115
BC/HAp/GO	70.146	4.22	1.055

### Biocompatibility

**Figure 10:**
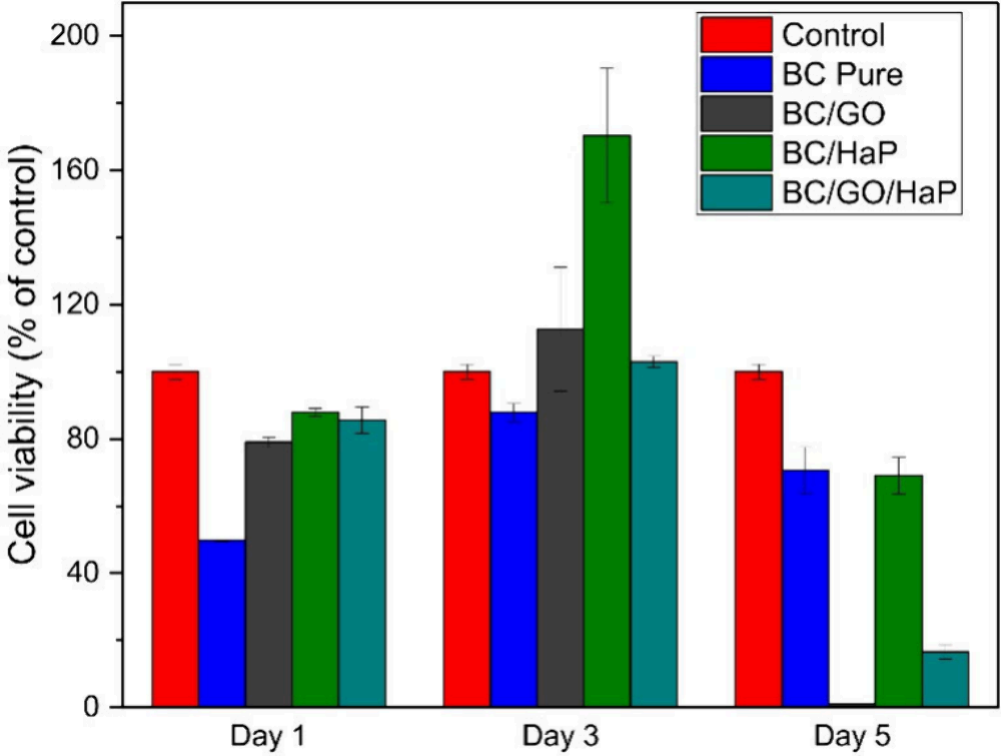
Cell proliferation result from MTT assay.

Cytotoxicity testing was performed for four different types of biomaterials to evaluate the proliferation of Saos-2 cells in the presence of a culture medium modified by the prepared scaffolds. The materials were pure BC, in situ synthesized BC/GO, in situ produced BC/HAp, and ex situ formed BC/HAp/GO (Method II). Figure 10 shows the cell viability of the materials compared with the control. The highest cell growth was registered by the addition of HAp in the BC scaffolds, which supplements the biocompatible nature of HAp. A 76% increase in cell viability on the first day and a 93.5% increase on the third day (from the respective cell viability figures of pure BC) were recorded. This high compatibility of HAp has been reported by previous studies, indicating the superb bioactivity of the Ca^2+^ and PO_4_^3–^ ions in terms of cell adhesion, spreading, and proliferation.^50−52^ Similarly, the addition of GO presented an improved cell viability compared to that of pure BC. A higher percentage of cell growth (79% and 112% of that of the control for the first and third days, respectively) was seen compared to that of BC. This phenomenon corresponds to the degree of biocompatibility and osteogenesis that GO enhances.^53^ The combined effect of GO and HAp also proved to be conducive (with cell viability values of 85.6% and 103% for first and third days, respectively). This indicates the biocompatible interaction between the Ca^2+^ ions of HAp and the hydroxy groups in GO. Moreover, the presence of GO in the composite likely induced apatite formation and cellular binding sites, promoting cell attachment.^54^ The improved surface roughness and porosity due to the properties of both GO and HAp particles, facilitated the delivery of oxygen and nutrients to the cells, promoting their proliferation.^32^ Given that the cell viability of the biocomposite BC/HAp/GO is well above 70%, it shows promise as a scaffold for bone tissue engineering applications.

It is noteworthy that, on the fifth day, the number of cells cultured in the presence of the modified media was lower compared to that on the third day. The reduction is most pronounced in BC/GO. This may be due to the following reasons. The first is the overgrowth of the cells and their detachment from the bottom of the wells and their subsequent loss during washing. The second is increased metabolic activity whereby byproducts such as lactate were formed. This possibly made the cell culture medium acidic, leading to a toxic environment for the cells.^55^ For full clarification of these possibilities, additional research is required with other cytotoxicity tests such as lactate dehydrogenaseassay as well as cytological, immunocytochemical, and molecular biological methods. On the other hand, the ex situ formed (Method III) BC/HAp/GO biocomposite showed the least biocompatibility and is not presented in Figure 10. A possible explanation could be complete destruction of the BC fibrils (as evidenced in Figure 6c) during homogenization.

## Conclusions

This study synthesized BC, HAp, and GO using alternative sustainable sources. Apple juice for the BC growth medium, egg shells as the calcium source of HAp, and coffee grounds as the carbon source for GO were utilized. The synthesized methodologies were non-energy-intensive and low-cost in design. The materials exhibited properties comparable to those synthesized conventionally. BC mats were structured in a microfibril format. The HAp particles showed sharp crystallinity with a Ca/P ratio of 1.72, closely matching the stoichiometric value of 1.67. GO particles exhibited the necessary functional groups found in commercially or conventionally produced GO. Their diffraction pattern indicated a more amorphous nature, due to the structural difference between a graphite and a biomass source. The BC/HAp/GO biocomposite was fabricated using in situ and ex situ methods, with the ex situ method involving the ultrasonication of BC mats in a HAp/GO solution yielding the most favorable results. The nanoparticles were observed to be physically cross-linked into and well-embedded in the BC matrix, without changing the crystalline nature of BC. The incorporation of GO increased the tensile strength (by about 25%) and Young’s modulus (by about 35%) of the pure BC mat. The addition of HAp particles enhanced bioactivity and supported the proliferation of human osteoblast-like cells (Saos-2) in vitro, highlighting the biocomposite’s biocompatibility. These findings suggest the possible application of such a biocomposite/biomaterial in bone tissue engineering. The study also underlines the effectiveness of using biomass/food waste as an alternative source of scaffold synthesis, contributing to an economically feasible and environmentally friendly approach to biomaterial production.
